# Supplementary material for: Controlling hot electron flux and catalytic selectivity with nanoscale metal-oxide interfaces
Source: Nat Commun. 2021 Jan 4;12:40. doi: 10.1038/s41467-020-20293-y (PMC7782808; doi:10.1038/s41467-020-20293-y)
Supplement: Supplementary file 1 — Supplementary Information [file 41467_2020_20293_MOESM1_ESM.pdf]

Supplementary Information

Controlling hot electron flux and catalytic selectivity with nanoscale metal-oxide interfaces

Si Woo Lee[1,2†], Jong Min Kim[3,4†], Woonghyeon Park[5†], Hyosun Lee[1], Gyu Rac Lee[3], Yousung Jung[5], Yeon Sik Jung[3]*, and Jeong Young Park[1,2]**

[1]Center for Nanomaterials and Chemical Reactions, Institute for Basic Science (IBS), Daejeon 34141, Republic of Korea

[2]Department of Chemistry, Korea Advanced Institute of Science and Technology (KAIST), Daejeon 34141, Republic of Korea

[3]Department of Materials Science and Engineering, Korea Advanced Institute of Science and Technology (KAIST), Daejeon 34141, Republic of Korea

[4]Materials Architecturing Research Center, Korea Institute of Science and Technology (KIST), Seoul 02792, Republic of Korea

[5]Department of Chemical and Biomolecular Engineering, Korea Advanced Institute of Science and Technology (KAIST), Daejeon 34141, Republic of Korea

*To whom correspondence should be addressed. E-mail: ysjn@kaist.ac.kr, ysjung@kaist.ac.kr, jeongypark@kaist.ac.kr
†These authors contributed equally to this work

Contents

Supplementary Notes

Supplementary Note 1 | Electrical properties of the Pt nanowires/TiO_2 catalytic nanodiodes.

Supplementary Note 2 | Maintained electrical stability of the catalytic nanodiodes under methanol oxidation reaction conditions.

Supplementary Note 3 | Negligible substrate effect on Pt film/TiO_2 catalyst.

Supplementary Note 4 | Change of chemicurrent yield according to the product formation.

Supplementary Note 5 | Comparison of activity and chemicurrent yield in Pt nanowires/TiO_2 and Pt film/TiO_2 under hydrogen oxidation reaction.

Supplementary Note 6 | Charge transfer from the methanol adsorbate to oxide supports by theoretical calculations.

Supplementary Note 7 | *I-V* curve, chemicurrent and reaction results on both the nanowires with width of 20 and 50 nm.

Supplementary Figures

Supplementary Fig. 1 | Fabrication process of Pt nanowires/TiO_2 catalytic nanodiode

Supplementary Fig. 2 | Morphology, XPS, and XRD characterization of the Pt nanowires deposited on TiO_2

Supplementary Fig. 3 | SEM images of Pt nanowires/TiO_2 with a width of 15 nm, 20 nm and 50 nm, and Pt film/TiO_2

Supplementary Fig. 4 | Reproducible chemicurrent results from changing partial pressure of methanol (1-4 Torr)

Supplementary Fig. 5 | Partial pressure dependence on chemicurrent obtained from Pt nanowires/TiO_2 catalytic nanodiode

Supplementary Fig. 6 | Maintained stability of the Pt nanowires/TiO_2 catalytic nanodiodes under reaction conditions

Supplementary Fig. 7 | *I-V* curve and Schottky barrier height of the Pt nanowires/TiO_2 Shottky nanodiode at a different partial pressure of methanol

Supplementary Fig. 8 | Catalytic activity and partial oxidation selectivity under 4 Torr of methanol and 760 Torr of O_2

Supplementary Fig. 9 | Plot of selectivity according to methanol conversion on Pt film/TiO_2 and Pt nanowires/TiO_2

Supplementary Fig. 10 | Comparison of activity and chemicurrent yield in Pt film/TiO_2 and Pt nanowires/TiO_2 measured at 333 K

Supplementary Fig. 11 | Comparison of activity and chemicurrent yield in Pt film/TiO_2 and Pt nanowires/TiO_2 measured at 343 K

Supplementary Fig. 12 | Current signal and chemicurrent yield on the Pt film/TiO_2 and Pt nanowires/TiO_2 catalytic nanodiode under hydrogen oxidation

Supplementary Fig. 13 | Catalytic activity and Arrhenius plots from the TOF and chemicurrent on the Pt film/TiO_2 and Pt nanowires/TiO_2 catalytic nanodiode under hydrogen oxidation

Supplementary Fig. 14 | TON and methanol conversion with reaction time measured under 4 Torr of methanol and 760 Torr of O_2 at 343 K

Supplementary Fig. 15 | The scheme, *I-V* curve and chemicurrent on TiO_2 nanowires/Pt film/TiO_2 inverse catalytic nanodiode

Supplementary Fig. 16 | Model structures of the Pt nanorod on TiO_2(110)

Supplementary Fig. 17 | Comparison of Lewis acidity of TiO_2 and SiO_2 support

Supplementary Fig. 18 | *I-V* curve, chemicurrent and catalytic activity on Pt nanowires (width 15 nm)/TiO_2 catalytic nanodiode

Supplementary Fig. 19 | Reproducible current signals of Pt nanowires (width 15 nm)/TiO_2 catalytic nanodiode obtained under different methanol partial pressure

Supplementary Fig. 20 | Remained electrical properties of the Pt nanowires (width 15 nm)/TiO_2 catalytic nanodiodes under chemical reaction conditions

Supplementary Fig. 21 | *I-V* curve, chemicurrent and catalytic activity on Pt nanowires (width 50 nm)/TiO_2 catalytic nanodiode

Supplementary Fig. 22 | Comparison of chemicurrent in all types of catalytic nanodiodes

Supplementary Fig. 23 | Plot of selectivity vs. methanol conversion under methanol 4 Torr and O_2 to 760 Torr

Supplementary Fig. 24 | Reaction scheme for methanol oxidation on Pt-TiO_2 interface

Supplementary Fig. 25 | Changed configuration of *CH_2O on Pt nanorod on TiO_2(110) and Pt(111)

Supplementary Fig. 26 | CO_2 adsorption energy on TiO_2(110) surface in Pt nanorod/TiO_2(110) model

Supplementary Tables

Supplementary Table 1 | Measured catalytic activity and calculated selectivity on Pt film/TiO_2.

Supplementary Table 2 Measured catalytic activity and calculated selectivity on Pt nanowires/TiO_2.

Supplementary Table 3 | Calculated Schottky barrier height for all Schottky nanodiodes by fitting *I-V* curve of each nanodevice to the thermionic emission theory.

Supplementary Table 4 | Calculated reaction energies and barriers for methanol oxidation on Pt nanorod structure on TiO_2(110).

Supplementary Table 5 | Calculated reaction energies and barriers for methanol oxidation on Pt nanorod structure on Pt(111).

Supplementary Notes

Supplementary Note 1 | Electrical properties of the Pt nanowires/TiO_2 catalytic nanodiodes.

To characterize the conductive properties (*e.g.,* Schottky barrier height, series resistance, and ideality factor of the nanodevice) of the fabricated Pt nanowires/TiO_2 Schottky nanodiodes, current-voltage (*I-V*) curves were obtained using a Keithley Instrumentation 2400 sourcemeter. According to the thermionic emission equation, a forward-biased current on the Schottky diode can be described by

$$I = I_S \left[\exp\left(\frac{q(V-IR_S)}{\eta k_B T}\right) - 1\right] \qquad (1)$$

$$I_S = FA^{*}T^2\, exp\left(-\frac{q\varphi_b}{k_B T}\right) \qquad (2)$$

where I_S is the saturation electric current, q is the elementary charge, R_S is the series resistance, F is the active area of the Schottky diode, A^{*} is the effective Richardson constant, T is the temperature, k_B is the Boltzmann constant, φ_b is the Schottky barrier height of the nanodiode, η is the ideality factor of the device[1]. For separating the bias voltage as a function of the diode current, eq S1 can be written after taking the logarithm as

$$V = \frac{\eta k_B T}{q} ln\left(\frac{I}{I_S}\right) + IR_S \qquad (3)$$

By fitting the measured *I–V* curve to this equation, a Schottky barrier height of 0.85 eV, ideality factor of 2.26, and series resistance of 550 ohms were obtained.

Supplementary Note 2 | Maintained electrical stability of the catalytic nanodiodes under methanol oxidation reaction conditions.

Similar to the experiments on the Pt film/TiO_2 catalytic nanodiode, we measured the electric currents as the partial pressure of methanol was changed for 1, 2, and 4 Torr at elevated reaction temperatures (Supplementary Fig. 4). As shown in Supplementary Fig. 5, the chemicurrent increased with the partial pressure of methanol, indicating that the trend of hot electron generation on the Pt nanowires/TiO_2 catalytic device with partial pressure is similar to the previous results on Pt film/TiO_2 nanodiode[2]. All the chemicurrent measurements were performed three times for reproducibility, and the stable steady-state currents appeared at all methanol pressure conditions, showing that the contact between the Pt nanowires and the TiO_2 support was well maintained during the chemical reaction. Furthermore, we confirmed the thermal stability of the catalytic nanodiode under methanol oxidation conditions at elevated reaction temperatures from the unchanged *I-V* curves before and after the chemical reaction three times (Supplementary Fig. 6). Thus, owing to the excess oxygen and low temperature conditions in this reaction, the rectification characteristics of the Schottky device were well maintained. Also, Supplementary Fig. 7 shows the *I-V* curve data and Schottky barrier height varying with a partial pressure of methanol, and the change in barrier height was negligible. In addition, in situ *I–V* curves during the reaction were relatively unchanged, and it can be assumed that the state of the Pt nanowires was maintained as a metallic state during the reaction since the *I–V* curve represents the state at the junction between the Pt nanowires and TiO_2.

Supplementary Note 3 | Negligible substrate effect on Pt film/TiO_2 catalyst.

As confirmed in the SEM image (Supplementary Fig. 3d), this 5 nm-thick Pt film was deposited on TiO_2 without any defects, so that bottom TiO_2 was not exposed to the gas-phase. That is, in the Pt film/TiO_2 catalyst, only the Pt surface participated in the catalytic reaction. In addition, to further confirm the substrate effect discussed by the reviewer, we additionally compared the selectivity after depositing the Pt film with the same thickness of 5 nm on a SiO_2 substrate instead of TiO_2. It was confirmed that the selectivity of Pt film/TiO_2 and Pt film/SiO_2 were similar in all methanol conversions, and thus it was confirmed once again that the 5 nm-thick Pt film had no substrate effect.

Supplementary Note 4 | Change of chemicurrent yield according to the product formation.

Since methanol oxidation is a multi-path reaction in which two products appear (*i.e.,* CO_2 and methyl formate), it is necessary to compare the hot electron excitation in catalytic nanodiodes of Pt nanowires/TiO_2 and Pt film/TiO_2 according to product formation. To this end, as the partial pressure of methanol was changed, we found a pressure range that produces only CO_2 by full oxidation (methanol 1 and 2 Torr) and a minimum methanol pressure (methanol 4 Torr) that additionally generates methyl formate by partial oxidation. Thus, the partial pressure of methanol was varied (methanol 1-4 Torr and O_2 to 760 Torr), and the results of TOF and chemicurrent yield for the Pt nanowires/TiO_2 and the Pt film/TiO_2 were compared. As shown in Supplementary Fig. 5, chemicurrent on Pt nanowires/TiO_2 was much higher under methanol 4 Torr than methanol 1 and 2 Torr with the O_2 to 760 Torr condition indicating that formation of methyl formate can significantly enhance the hot electron generation. These results show that our previous finding of the influence of selectivity to hot electron excitation also applies to the Pt nanowires/TiO_2 catalytic nanodiode[2]. When comparing the total TOF values under all partial pressure conditions of methanol, the values were similar in Pt nanowires/TiO_2 and Pt film/TiO_2 (Supplementary Fig. 10a). As shown in Supplementary Fig. 10b, under methanol 1 and 2 Torr conditions, no significant difference in the chemicurrent yield was found for Pt nanowires/TiO_2 and the Pt film/TiO_2 nanodiode. As mentioned earlier, because of the excess oxygen conditions in the 1 and 2 Torr environments of methanol, methyl formate was not produced, and only CO_2 was produced; thus, the chemicurrent yield was affected only by the production of CO_2. Therefore, in these reaction environments (methanol 1-2 Torr and O_2 to 760 Torr), the TOF values of the Pt nanowires/TiO_2 and the Pt film/TiO_2 were similar, resulting in similar chemicurrent yields. However, in the methanol 4 Torr environment, Pt nanowires supported on TiO_2 showed higher chemicurrent yield than Pt film. In this increased methanol environment, a partial oxidation reaction took place, methyl formate formed, and the chemicurrent yield was determined by partial oxidation selectivity. Thus, the Pt nanowires showed higher selectivity due to the Pt-TiO_2 interface, resulting in higher chemicurrent yield. Similar trends of the partial pressure dependence of chemicurrent yield were also observed at different reaction temperatures (Supplementary Fig. 11). Therefore, these findings suggest that the enhanced selectivity toward methyl formate formation on the Pt nanowires/TiO_2 interface can significantly change the dynamics of hot electron excitation from chemical reactions.

Furthermore, we conclude that the efficiency of hot electron generation was affected by the metal-oxide interface in a multi-path reaction (*i.e.,* production of both methyl formate and CO_2 under methanol 4 Torr and O_2 to 760 Torr), not in a one-path reaction (*i.e.,* only CO_2 was generated under methanol 1-2 Torr and O_2 to 760 Torr). Therefore, through this change of the chemicurrent yield according to partial pressure of methanol, it can be seen that chemicurrent yield, which represents the efficiency of hot electron excitation, was greatly influenced by selectivity to methyl formate, not reactivity.

Supplementary Note 5 | Comparison of activity and chemicurrent yield in Pt nanowires/TiO_2 and Pt film/TiO_2 under hydrogen oxidation reaction.

Similar to the reaction environment of methanol oxidation (*i.e.,* methanol 4 Torr and O_2 to 760 Torr), the catalytic reactions were conducted in hydrogen 4 Torr and oxygen to 760 Torr. As shown in Supplementary Fig. 12, the chemicurrents excited by hydrogen oxidation in Pt nanowires and Pt films were similar. The chemicurrent yield values were also similar for two catalytic nanodiodes, since the hydrogen oxidation reaction was the one-path reaction that differed from the methanol oxidation environment in which the selectivity existed (Supplementary Fig. 12c). From these results, we confirmed that chemicurrent yield, the efficiency of hot electron generation, was attributed to selectivity, not reactivity. Furthermore, when TOF values were compared, the values were similar in both catalysts, and the activation energy values were almost the same for Pt nanowires/TiO_2 and Pt films/TiO_2; thus, we found that the effect of the Pt-TiO_2 interface was negligible in the hydrogen oxidation reaction, the one-path reaction in which only water was formed (Supplementary Fig. 13a-c). In the hydrogen oxidation reaction in which selectivity did not actually appear (*i.e.,* only reactivity), the change in reactivity and chemicurrent yield by the Pt-TiO_2 interface did not appear. Therefore, from these control experiments, it can be concluded that selectivity has a much greater effect than reactivity in determining chemicurrent yield.

Supplementary Note 6 | Charge transfer from the methanol adsorbate to oxide supports by theoretical calculations.

To reveal the Lewis acid/base properties of TiO_2 and SiO_2 supports under the methanol oxidation reaction environment, we investigated the charge transfer from the methanol adsorbate to the oxide support surface by using density functional theory (DFT) calculation when reactant methanol molecule was adsorbed on TiO_2 and SiO_2 support. In the chemistry of Lewis acid/base properties on oxide surfaces, it is a Lewis acid when electron charge is received and a Lewis base when electron charge is lost[3]. The charge transfer by the interaction between adsorbate molecule-oxide was investigated using the Bader charge analysis simulation method, which is commonly used to examine the Lewis acidity of oxide supports[3-5]. As in the model designed for DFT calculation on the Au-TiO_2 interface in the Yates group[6,7], three atomic layers of Pt nanorod bonded on top of the rutile TiO_2(110) and SiO_2 surface was used to model the Pt nanowires/TiO_2 and Pt nanowires/SiO_2. We used the interfacial structure of a Pt-oxide support, as shown in Supplementary Fig. 16, to calculate the charge transfer between methanol adsorbate and oxide surfaces.

Supplementary Note 7 | *I-V* curve, chemicurrent and reaction results on both the nanowires with width of 20 and 50 nm.

Based on the *I-V* curve obtained from the Pt nanowires (width 15 nm)/TiO_2 catalytic nanodiode fabricated in this way, fitting to the thermionic emission equation showed a slightly higher barrier height than the height of the previous Pt nanowire-based nanodiode (Schottky barrier height = 1.02 eV, as shown in Supplementary Fig. 18a). Under methanol 4 Torr and O_2 to 760 Torr, the TOF, selectivity, and chemicurrent (*i.e.,* the hot electron generation) could all be detected on the Pt nanowires (width 15 nm)/TiO_2 catalytic nanodiode (Supplementary Fig. 18b-c). Moreover, similar to the trend in the Pt nanowires with width 20 nm, the reproducible reaction electric currents varying with a partial pressure of methanol for 1, 2, and 4 Torr for three cycles were measured, and the chemicurrent increased with the partial pressure of methanol (Supplementary Fig. 19). Furthermore, the device was stable through the unchanged *I-V* curve after the catalytic reaction (Supplementary Fig. 20).

In Pt nanowires (width 50 nm)/TiO_2 catalytic nanodiode, a system that increases the width of nanowires, *I-V* curve measurement showed that the Schottky nanodevice was well-fabricated with a Schottky barrier height of 0.89 eV (Supplementary Fig. 21a). As in the experiments with Pt nanowires with width 15 nm and 20 nm, the steady-state chemicurrent could be measured on the fabricated Pt nanowires (width 50 nm)/TiO_2 catalytic nanodiode under methanol 4 Torr and oxygen to 760 Torr, and the thermal stability of the nanodevice under reaction conditions was also confirmed by checking the unchanged electrical properties (Supplementary Fig. 21b and 21c). In addition, the selectivity toward methyl formate was measured to confirm the metal-oxide interface effect at increased width of Pt nanowires (Supplementary Fig. 21d).

Supplementary Figures

1. Fabrication process of Pt nanowires/TiO_2 catalytic nanodiode.

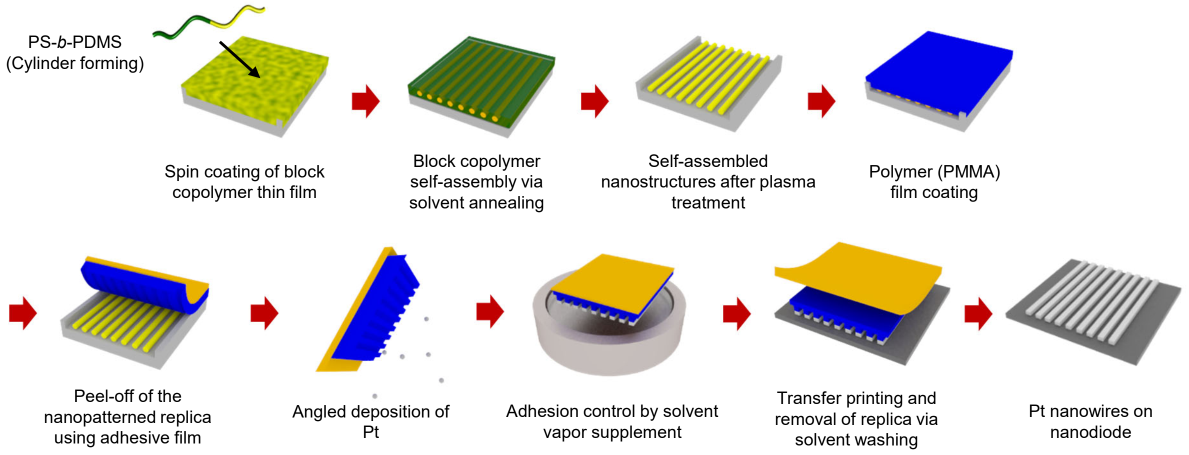

Supplementary Fig. 1 | Schematic illustration for solvent-assisted nanotransfer printing (s-nTP) process with using a master mold for depositing Pt nanowire arrays on TiO_2 support for constructing Pt nanowires/TiO_2 catalytic nanodiode..

2. Morphology, XPS, and XRD characterization of the Pt nanowires deposited on TiO_2.

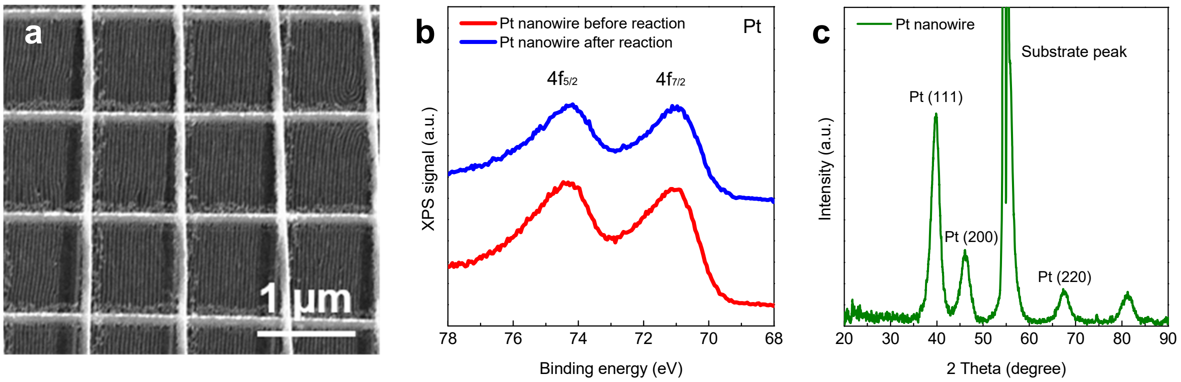

Supplementary Fig. 2 | a SEM image of Pt nanowire arrays on TiO_2 fabricated by master template. Bulk Au wire with the size of 200 nm was printed on the Pt nanowire arrays for preventing the electrical short by the defect of Pt nanowires. **b** XPS spectra for Pt 4f measured on the Pt nanowires/TiO_2 catalytic nanodiode before and after methanol oxidation reaction. **c** XRD patterns of the Pt nanowires with the period of 50 nm deposited on the silicon wafer.

3. SEM images of Pt nanowires/TiO_2 with the period of 50 nm and 35 nm.

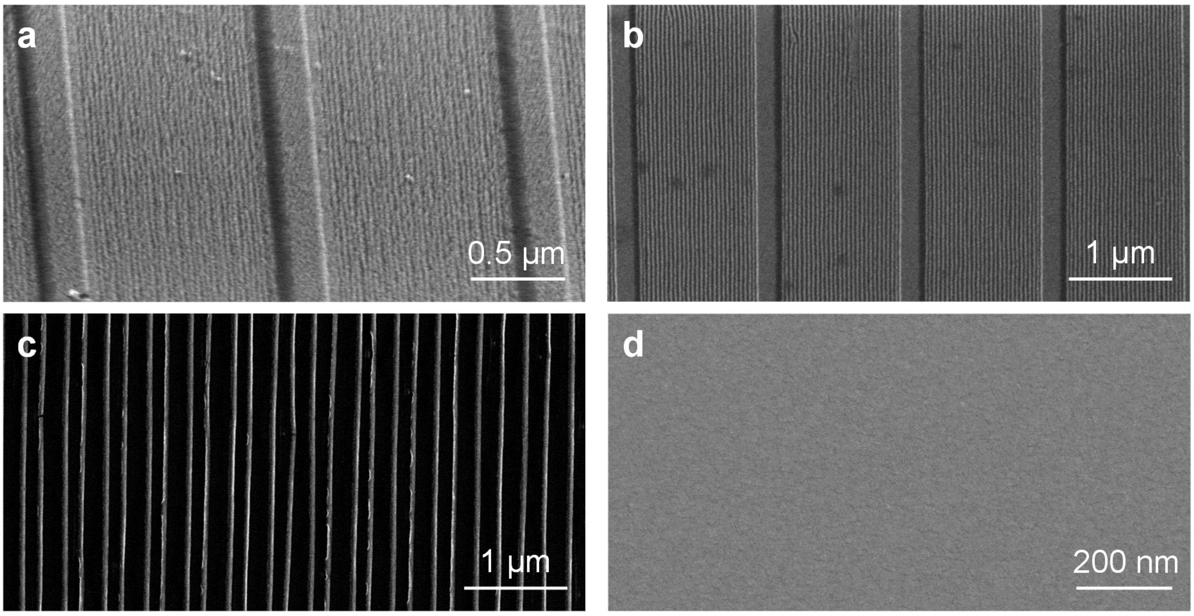

Supplementary Fig. 3 | SEM images of Pt nanowire arrays deposited on TiO_2 support using a self-assembled block copolymer with width of **a** 15 nm, **b** 20 nm, **c** 50 nm, and **d** Pt film.

4. Reproducible chemicurrent results from changing partial pressure of methanol (1-4 Torr).

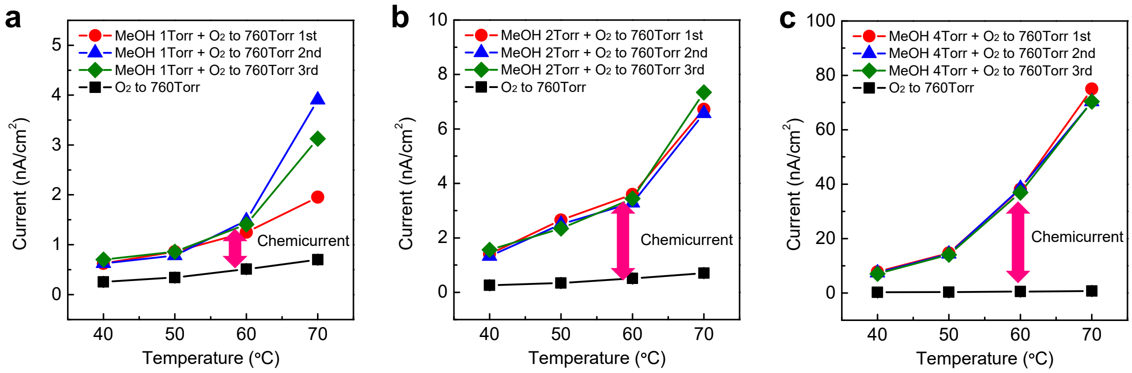

Supplementary Fig. 4 | Current measurement by ammeter under methanol oxidation on Pt nanowires/TiO_2 catalytic nanodiode under pressures of **a** 1 Torr, **b** 2 Torr and **c** 4 Torr methanol in 760 Torr of O_2 with elevated reaction temperature.

5. Partial pressure dependence on chemicurrent obtained from Pt nanowires/TiO_2 catalytic nanodiode.

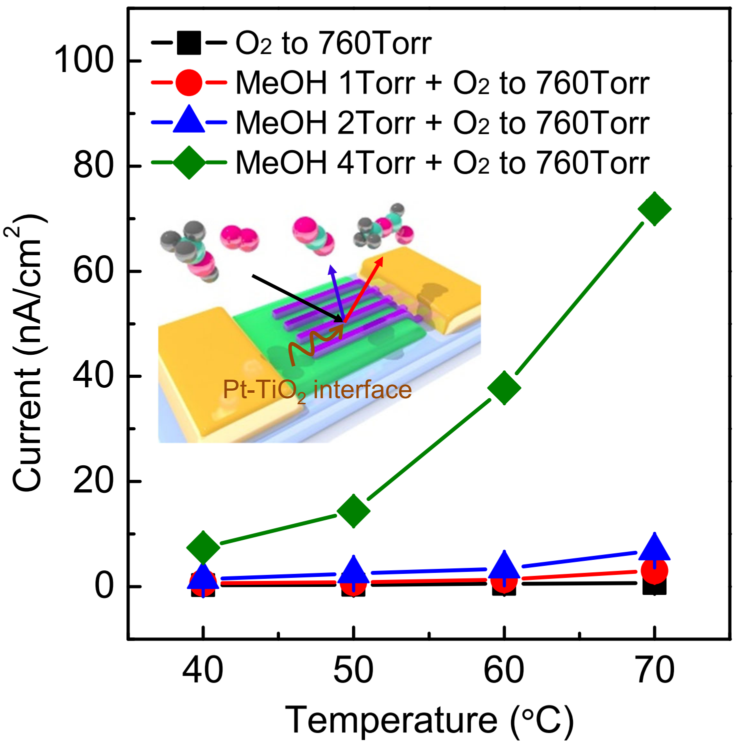

Supplementary Fig. 5 | Measurements of net chemicurrent generated by catalytic methanol oxidation on Pt nanowires/TiO_2 Schottky nanodiode varying with the partial pressure of methanol (1-4 Torr) in 760 Torr of O_2 at different temperatures, showing the methanol partial pressure dependence on hot electron generation.

6. Maintained stability of the Pt nanowires/TiO_2 catalytic nanodiodes under reaction conditions.

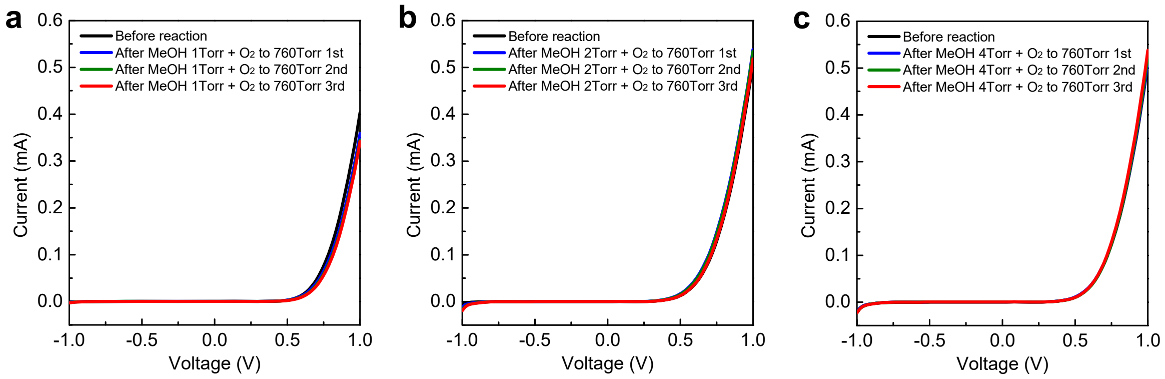

Supplementary Fig. 6 | *I*–*V* curves of the Pt nanowires/TiO_2 catalytic nanodiode obtained before and after three cycles of methanol oxidation during pressures of **a** 1, **b** 2, and **c** 4 Torr of methanol with O_2 to equal 760 Torr. Electrical properties of the nanodiodes were maintained for every three cycles of reaction.

7. *I-V* curve and Schottky barrier height of the Pt nanowires/TiO_2 Shottky nanodiode at different partial pressures of methanol.

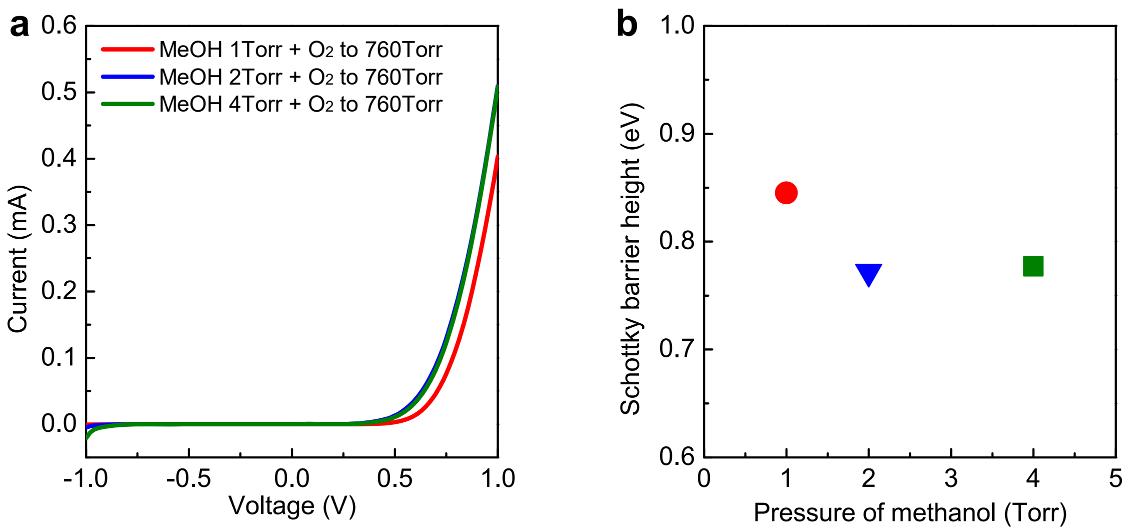

Supplementary Fig. 7 | a *I-V* curves measured from the Pt nanowires/TiO_2 catalytic nanodiode under reaction conditions with different partial pressures of methanol. **b** Schottky barrier height with different pressures of methanol.

8. Catalytic activity and partial oxidation selectivity under 4 Torr of methanol and 760 Torr of O_2.

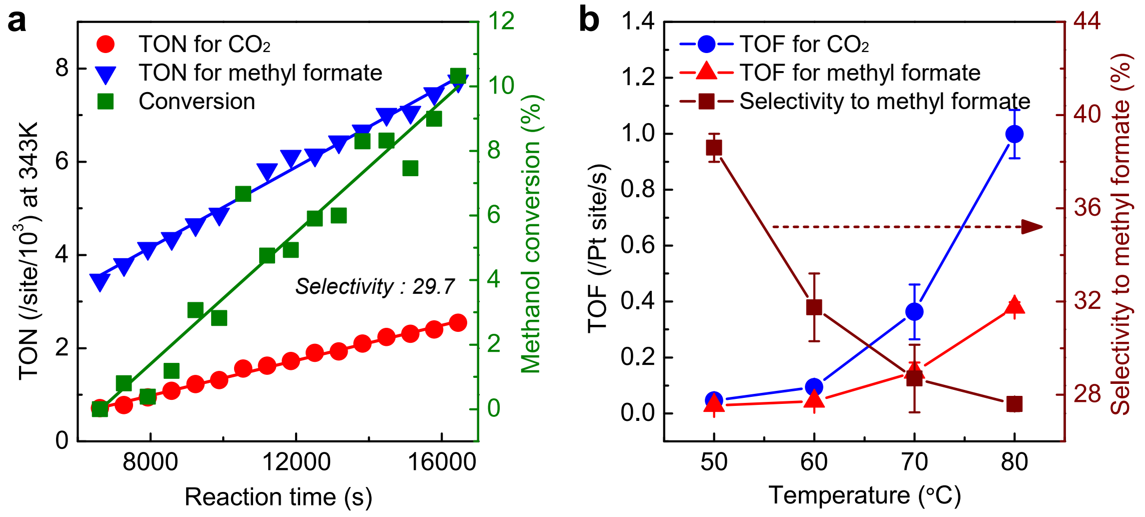

Supplementary Fig. 8 | a TON with reaction time for Pt nanowires/TiO_2 catalysts measured under 4 Torr of methanol and 760 Torr of O_2 at 343 K. Each TOF can be obtained from the slope in the graph, and the selectivity to methyl formate production was obtained from the ratio of each TOF value. All TOF and selectivity were obtained in methanol conversion of less than 10 %. **b** TOF for CO_2 and methyl formate formation and partial oxidation selectivity of Pt nanowires/TiO_2 catalysts during methanol oxidation with 4 Torr of methanol and 760 Torr of O_2 at different temperatures.

9. Plot of selectivity according to methanol conversion on Pt film/TiO_2 and Pt nanowires/TiO_2.

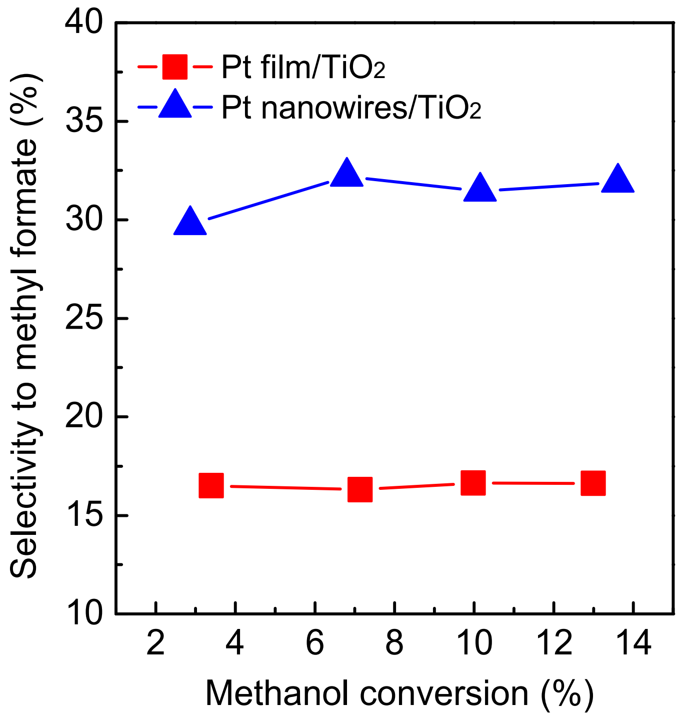

Supplementary Fig. 9 | Plot of selectivity according to methanol conversion on the catalytic nanodiodes of Pt film/TiO_2 and Pt nanowires/TiO_2 measured at 353 K.

10. Comparison of activity and chemicurrent yield in Pt film/TiO_2 and Pt nanowires/TiO_2 meausred at 333 K.

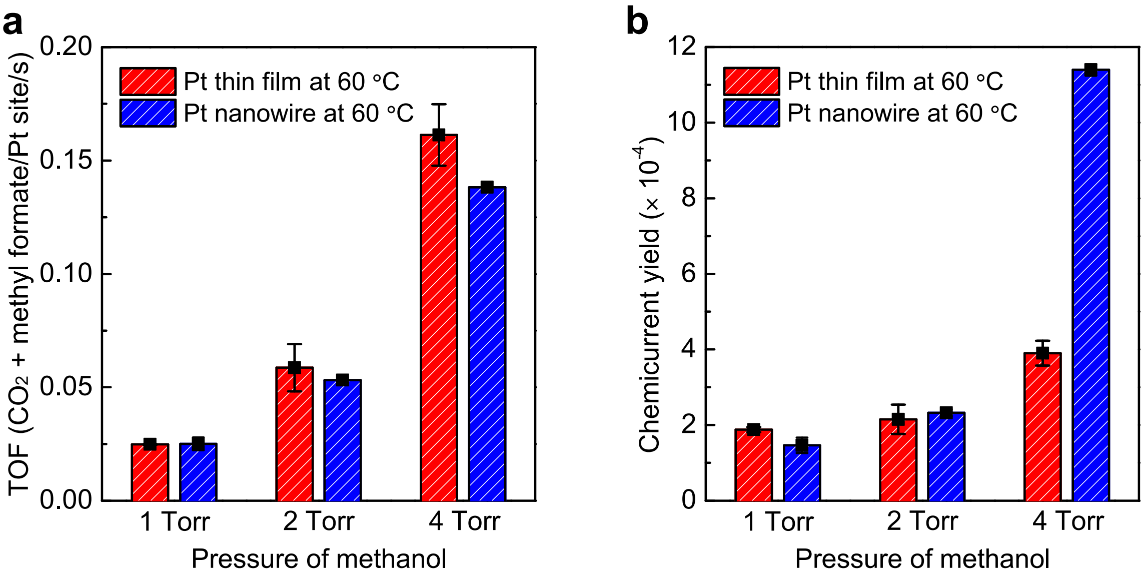

Supplementary Fig. 10 | a Plot of TOF for production of CO_2 and methyl formate and selectivity to methyl formate as a function of partial pressures of methanol measured on both the Pt film/TiO_2 and Pt nanowires/TiO_2 catalysts at 333 K. **b** The plot of chemicurrent yield as a function of partial pressures of methanol detected on both the Pt film/TiO_2 and Pt nanowires/TiO_2 catalytic nanodiodes. Error bars are ± sd.

11. Comparison of activity and chemicurrent yield in Pt film/TiO_2 and Pt nanowires/TiO_2 measured at 343 K.

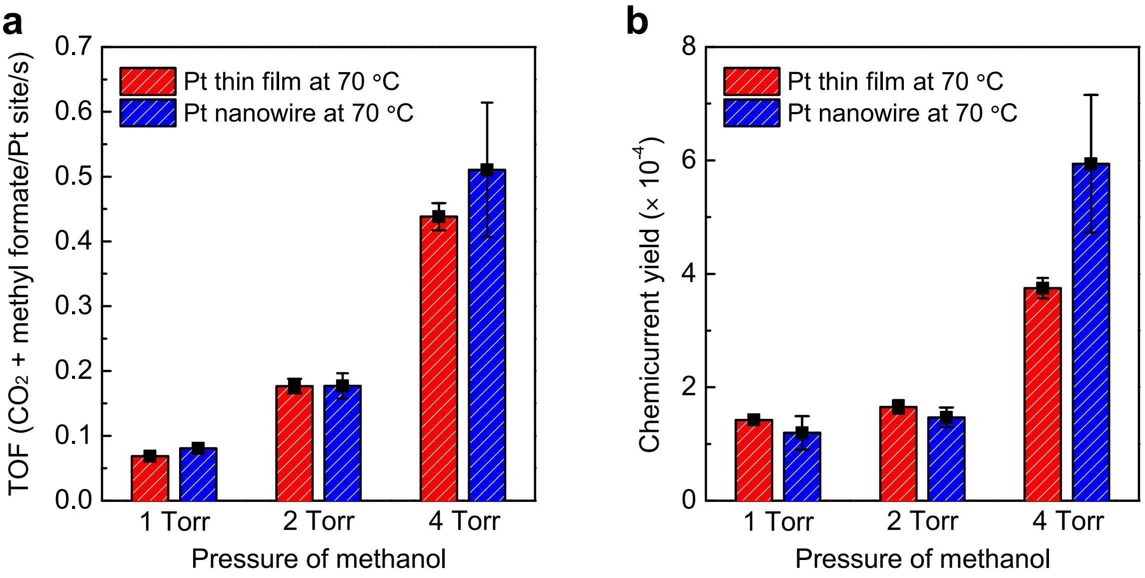

Supplementary Fig. 11 | a Plot of TOF for production of CO_2 and methyl formate and selectivity to methyl formate as a function of partial pressures of methanol measured on both the Pt film/TiO_2 and Pt nanowires/TiO_2 catalysts at 343 K. **b** The plot of chemicurrent yield as a function of partial pressures of methanol detected on both the Pt film/TiO_2 and Pt nanowires/TiO_2 catalytic nanodiodes. Error bars are ± sd.

12. Current signal and chemicurrent yield on the Pt film/TiO_2 and Pt nanowires/TiO_2 catalytic nanodiode under hydrogen oxidation.

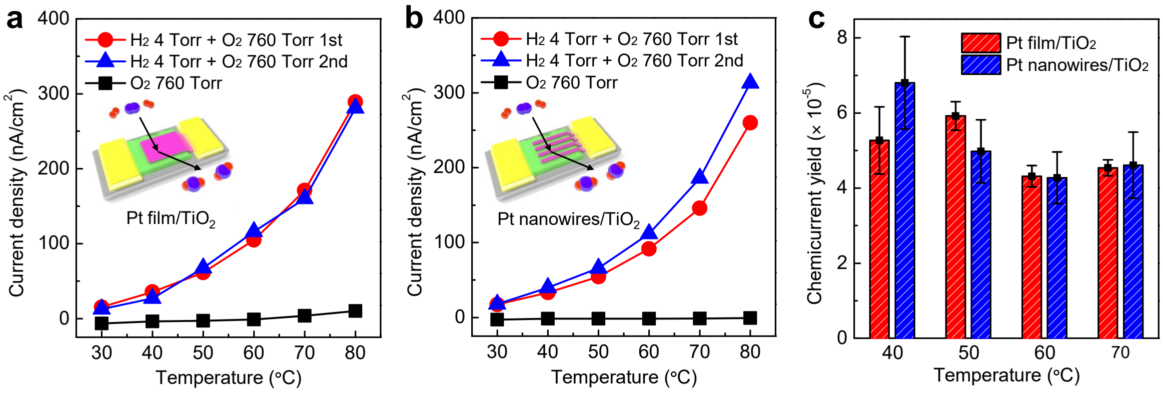

Supplementary Fig. 12 | Current measured on **a** Pt nanofilm/TiO_2 and **b** Pt nanowires/TiO_2 catalytic nanodiode in 4 Torr of hydrogen and 760 Torr of O_2 with elevated reaction temperature. **c** Chemicurrent yield on Pt film/TiO_2 and Pt nanowires/TiO_2 catalytic nanodiode with different reaction temperatures under hydrogen oxidation. Error bars are ± sd.

13. Catalytic activity and Arrhenius plots from the TOF and chemicurrent on the Pt film/TiO_2 and Pt nanowires/TiO_2 catalytic nanodiode under hydrogen oxidation.

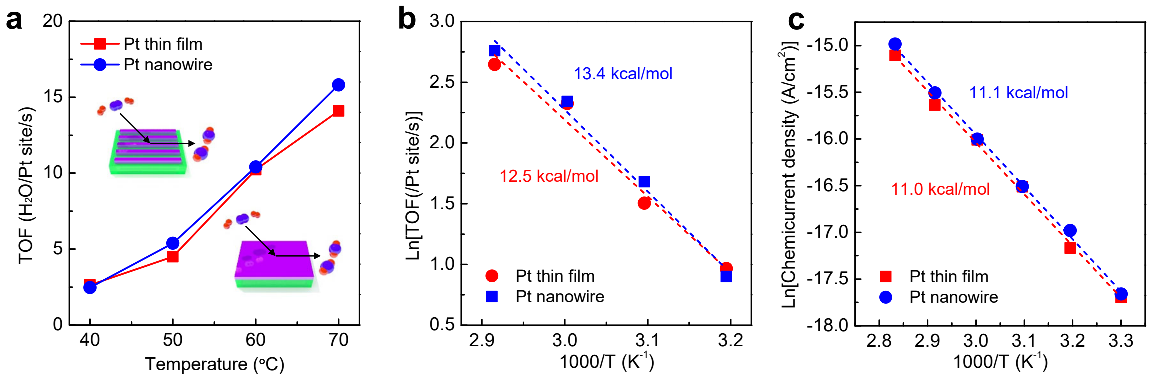

Supplementary Fig. 13 | a TOF for production of H_2O under 4 Torr of hydrogen and 760 Torr of O_2 on the Pt film/TiO_2 and Pt nanowires/TiO_2 with different reaction temperatures. Arrhenius plots obtained from measurement of **b** TOF and **c** chemicurrent, detected by the Pt film/TiO_2 and Pt nanowires/TiO_2 catalytic nanodiode under 4 Torr of hydrogen and 760 Torr of O_2.

14. TON and methanol conversion with reaction time.

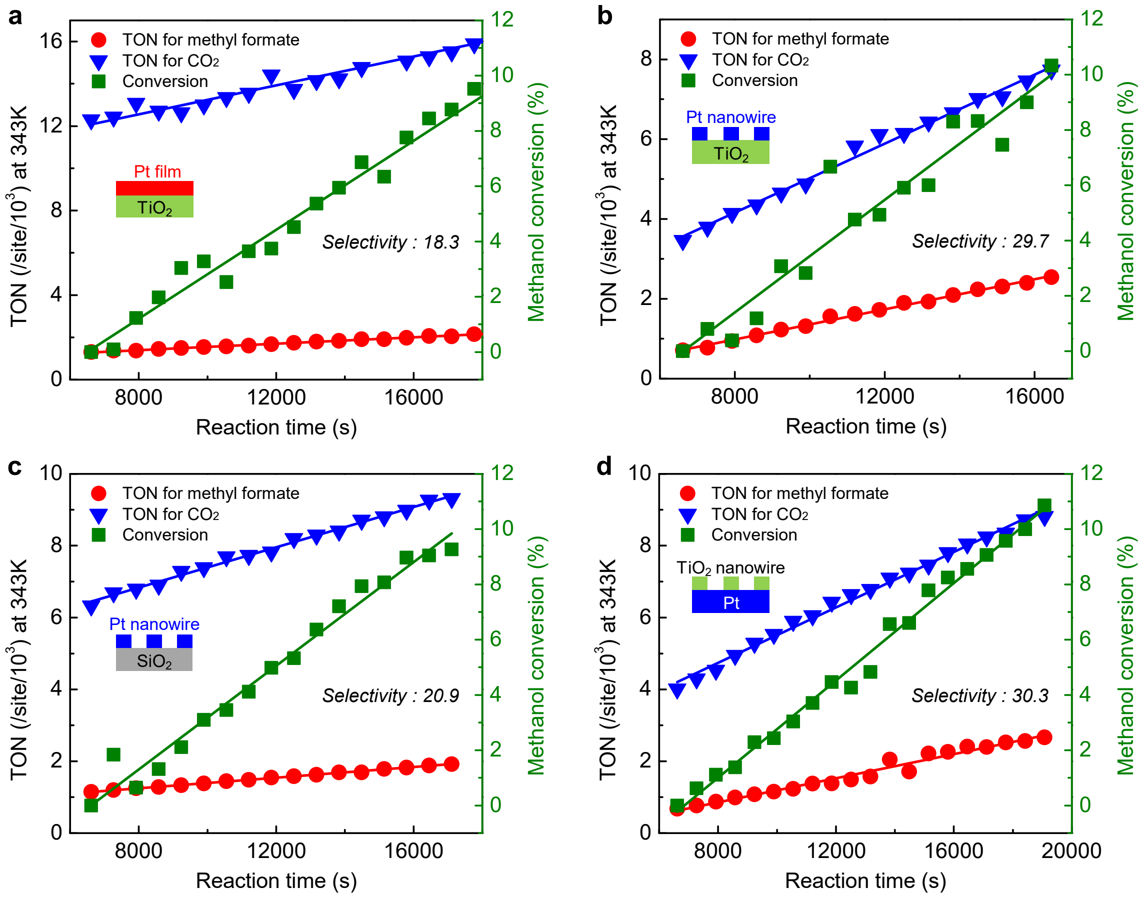

Supplementary Fig. 14 | TON and methanol conversion with reaction time measured under 4 Torr of methanol and 760 Torr of O_2 at 343 K for **a** Pt film/TiO_2, **b** Pt nanowires (width 20 nm)/TiO_2, **c** Pt nanowires (width 20 nm)/SiO_2, **d** Pt nanowires (width 50 nm)/TiO_2 catalysts. All TOFs and selectivity were compared in a region with a methanol conversion of less than 10%.

15. Scheme, *I-V* curve and chemicurrent on TiO_2 nanowires/Pt film/TiO_2 inverse catalytic nanodiode.

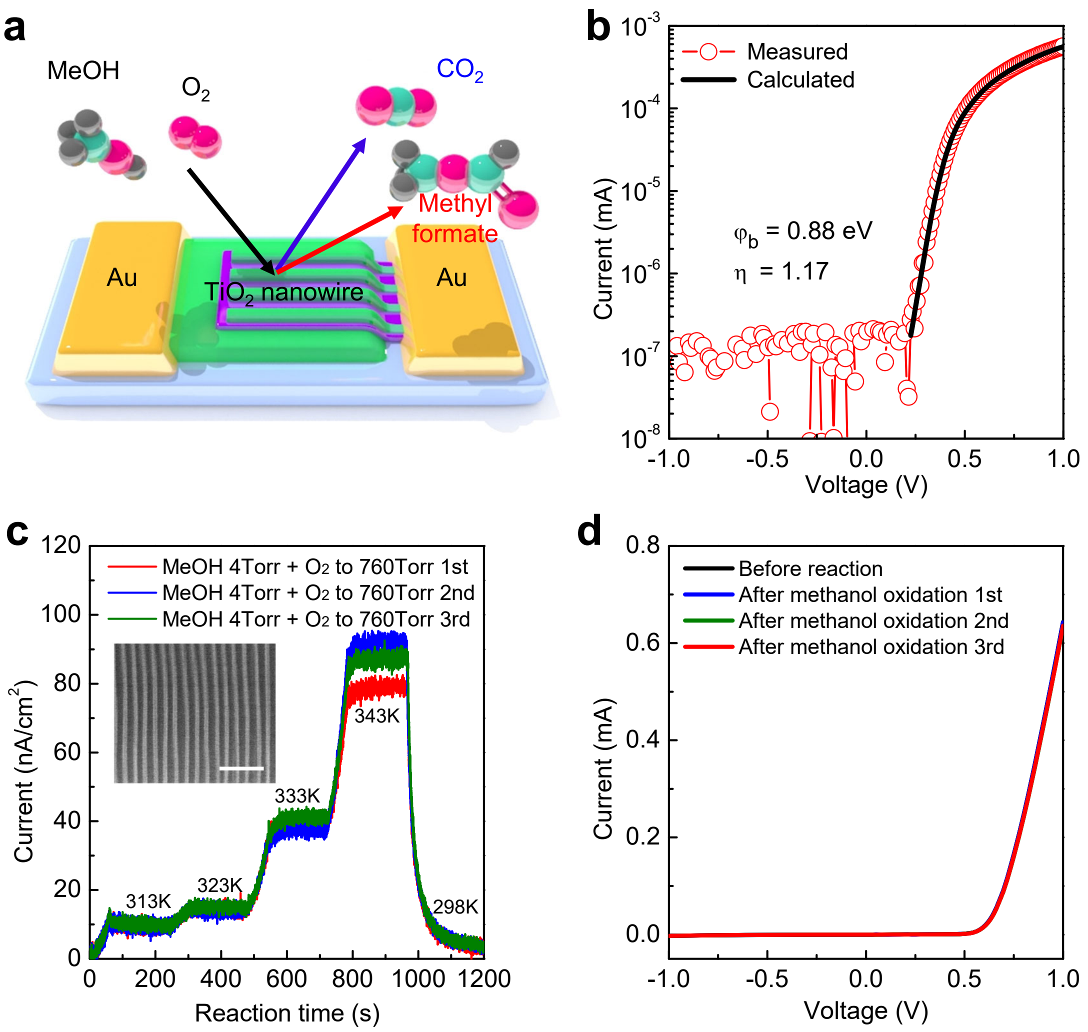

Supplementary Fig. 15 | a Schematic drawing of methanol oxidation at TiO_2-Pt interfical sites formed from TiO_2 nanowires/Pt film/TiO_2 inverse catalytic nanodiode. **b** *I-V* characteristics obtained from the TiO_2 nanowires/Pt film/TiO_2 inverse catalytic nanodiode, which is obtained for a Schottky barrier height of 0.88 eV, and an ideality factor of 1.17, respectively. **c** Chemicurrent on a TiO_2 nanowires/Pt film/TiO_2 inverse catalytic nanodiode measured under 4 Torr of methanol with O_2 to 760 Torr with elevated reaction temperature. Inset shows SEM image of TiO_2 nanowire arrays deposited on Pt film with a width of 20 nm and a pitch of 50 nm. Scale bars are 200 nm. **d** *I-V* curves of the TiO_2 nanowires/Pt film/TiO_2 inverse catalytic nanodiode before and after three cycles of catalytic reaction, indicating that the conductive properties of the inverse catalytic nanodiode were maintained.

16. **Model structures of the Pt nanorod on TiO_2(110)**

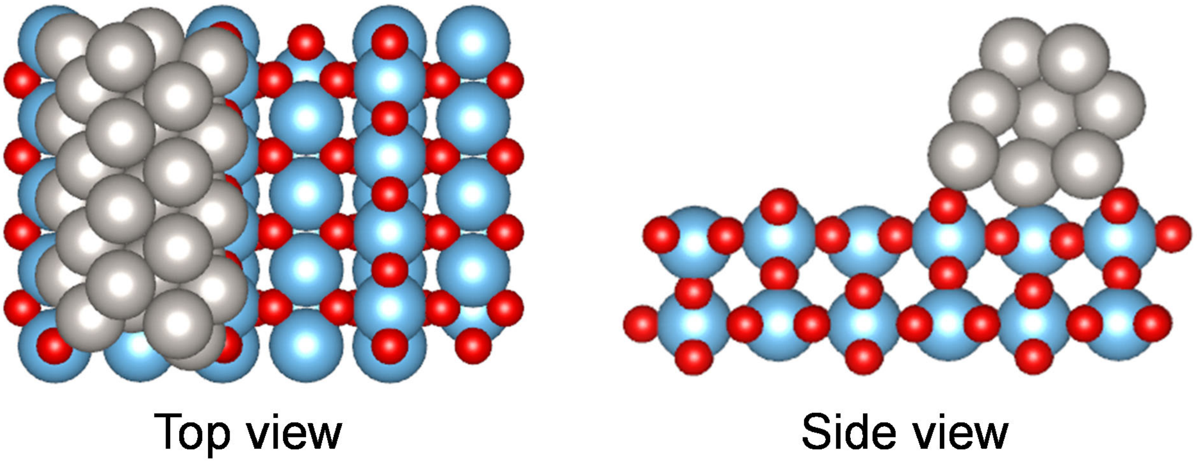

Supplementary Fig. 16 | DFT calculation models for methanol oxidation on Pt nanorod structure on TiO_2(110). The colors of each atom in the model are Pt (grey), Ti (light blue), O (red).

17. **Comparison of Lewis acidity of TiO_2 and SiO_2 support**

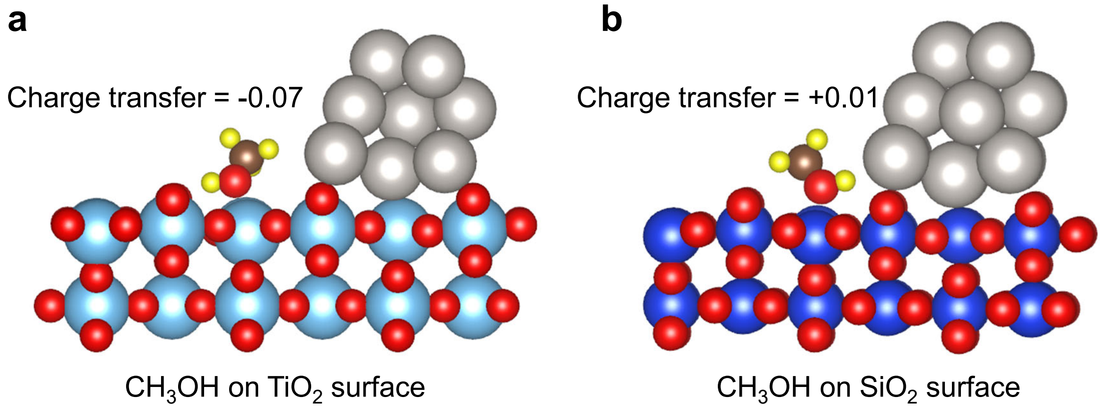

Supplementary Fig. 17 | Charge transfer from methanol adsorbate to oxide surfaces calculated by using Bader charge analysis simulation: **a** Pt nanorod/TiO_2, and **b** Pt nanorod/SiO_2.

18. *I-V* curve, chemicurrent and catalytic activity on Pt nanowires (width 15 nm)/TiO_2 catalytic nanodiode.

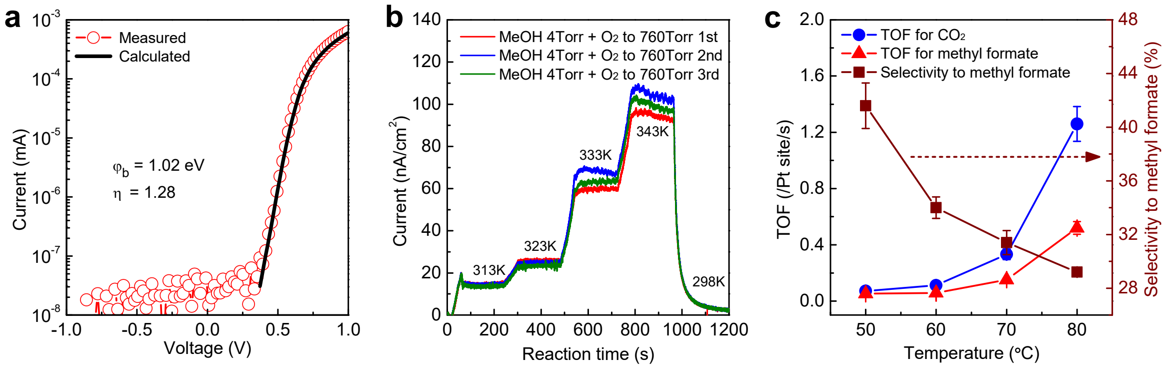

Supplementary Fig. 18 | a *I-V* characteristics obtained from the Pt nanowires (width 15 nm)/TiO_2 catalytic nanodiode, which is obtained for a Schottky barrier height of 1.02 eV, and an ideality factor of 1.28, respectively. **b** Chemicurrent on Pt nanowires (width 15 nm)/TiO_2 catalytic nanodiode measured under 4 Torr of methanol with O_2 to 760 Torr with elevated reaction temperature. **c** TOF for production of CO_2 and methyl formate and selectivity to methyl formate on Pt nanowires (width 15 nm)/TiO_2 during 4 Torr of methanol and 760 Torr of O_2 with different reaction temperatures.

19. Reproducible current signals of Pt nanowires (width 15 nm)/TiO_2 catalytic nanodiode obtained under different methanol partial pressures.

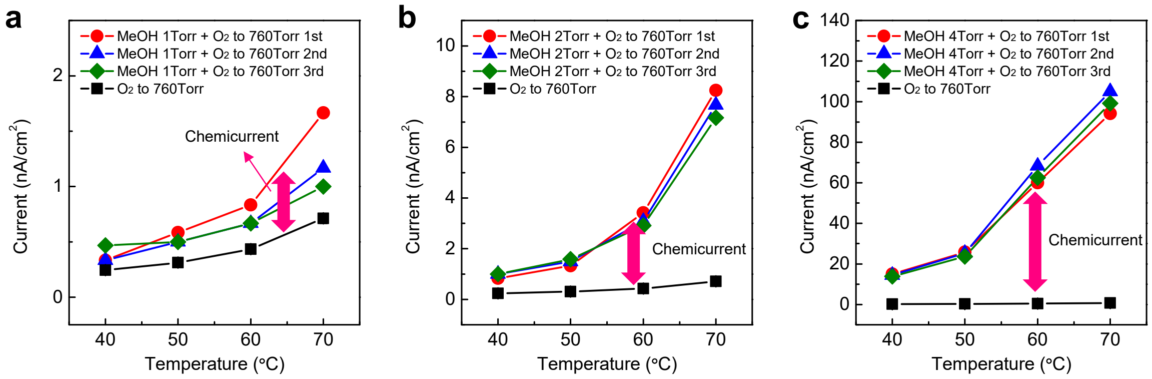

Supplementary Fig. 19 | Plot of measured current as a function of temperature under methanol oxidation on Pt nanowires (pich 35 nm)/TiO_2 catalytic nanodiode under pressure of **a** 1 Torr, **b** 2 Torr and **c** 4 Torr methanol and 760 Torr of O_2.

20. Remained electrical properties of the Pt nanowires (width 15 nm)/TiO_2 catalytic nanodiodes under chemical reaction conditions.

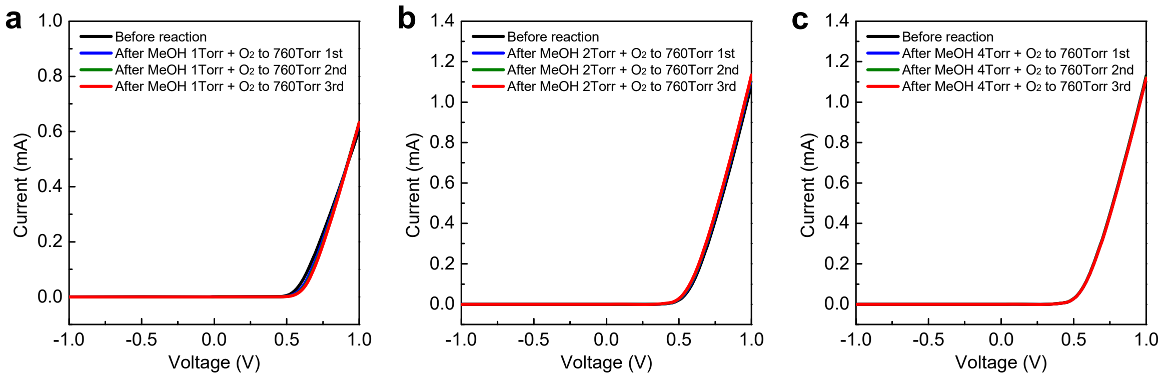

Supplementary Fig. 20 | *I–V* curves of the Pt nanowires (width 15 nm)/TiO_2 catalytic nanodiode obtained before and after three cycles of methanol oxidation during pressures of **a** 1, **b** 2, and **c** 4 Torr of methanol with O_2 to equal 760 Torr.

21. *I-V* curve, chemicurrent and catalytic activity on Pt nanowires (width 50 nm)/TiO_2 catalytic nanodiode.

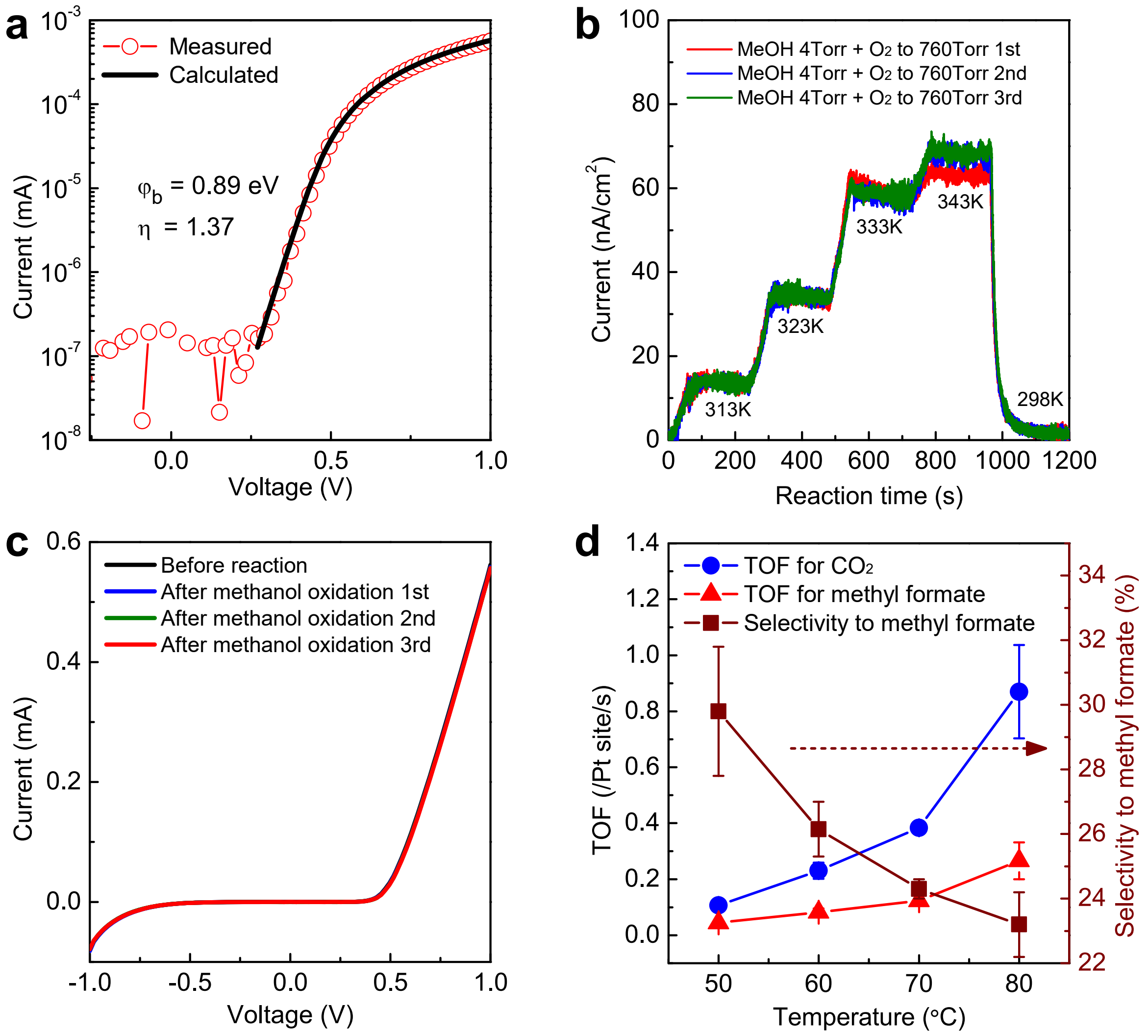

Supplementary Fig. 21 | a *I-V* characteristics obtained from the Pt nanowires (width 50 nm)/TiO_2 catalytic nanodiode, obtained for a Schottky barrier height of 0.89 eV and an ideality factor of 1.37, respectively. **b** Chemicurrent on Pt nanowires (width 50 nm)/TiO_2 catalytic nanodiode measured under 4 Torr of methanol with O_2 to 760 Torr with elevated reaction temperature. **c** *I-V* curves of the Pt nanowires (width 50 nm)/TiO_2 catalytic nanodiode before and after three cycles of catalytic reaction. **d** TOF for production of CO_2 and methyl formate and selectivity to methyl formate on Pt nanowires (width 50 nm)/TiO_2 during 4 Torr of methanol and 760 Torr of O_2 with different reaction temperatures.

22. Comparison of chemicurrent in all types of catalytic nanodiodes.

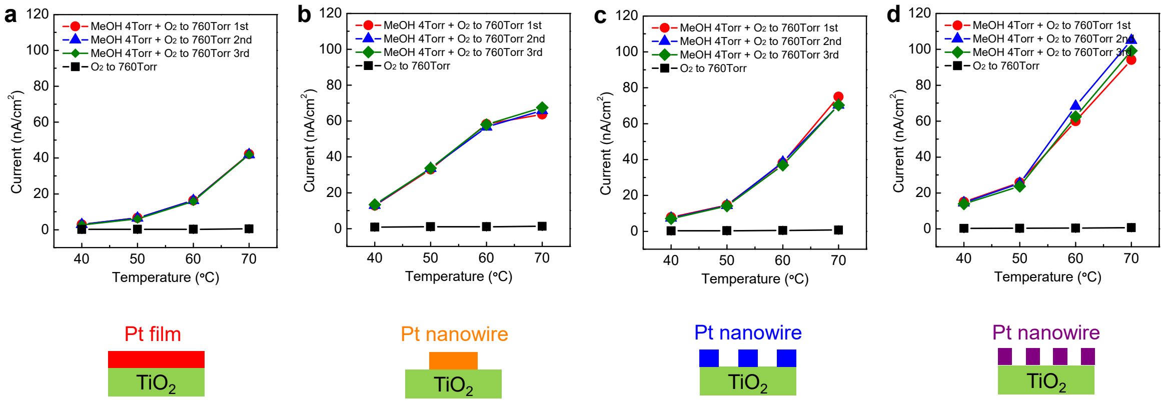

Supplementary Fig. 22 | Current measurement in 4 Torr of methanol and 760 Torr of O_2 on **a** Pt film/TiO_2, **b** Pt nanowires (width 50 nm)/TiO_2, **c** Pt nanowires (width 20 nm)/TiO_2 and **d** Pt nanowires (width 15 nm)/TiO_2 catalytic nanodiode with elevated reaction temperature.

23. Plot of selectivity vs. methanol conversion under methanol 4 Torr and O_2 to 760 Torr.

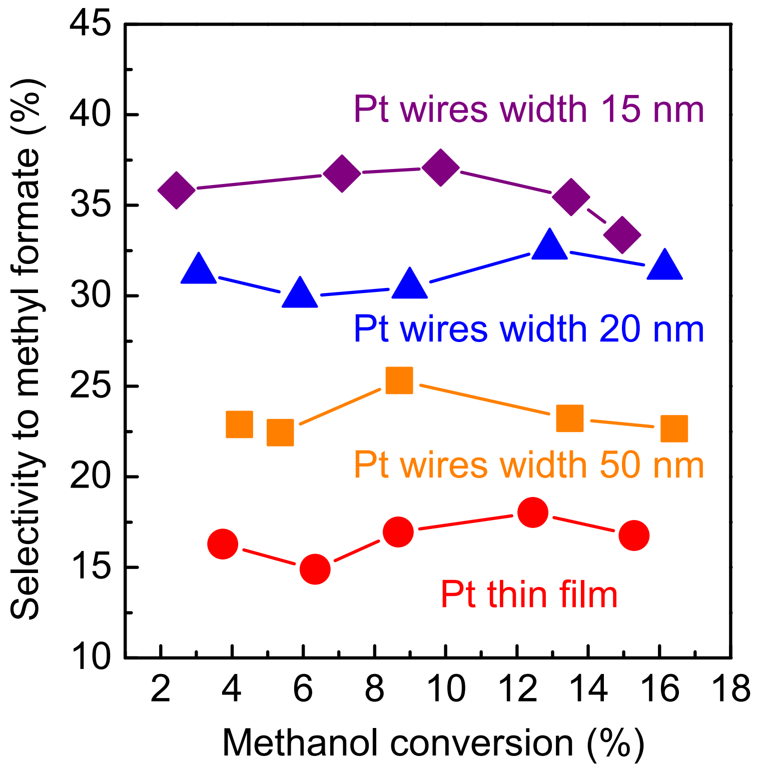

Supplementary Fig. 23 | Plot of selectivity according to methanol conversion on the catalytic nanodiodes of Pt film/TiO_2, Pt nanowires (width 50 nm)/TiO_2, Pt nanowires (width 20 nm)/TiO_2, and Pt nanowires (width 15 nm)/TiO_2 measured at 343 K..

24. Reaction scheme for methanol oxidation on Pt-TiO_2 interface

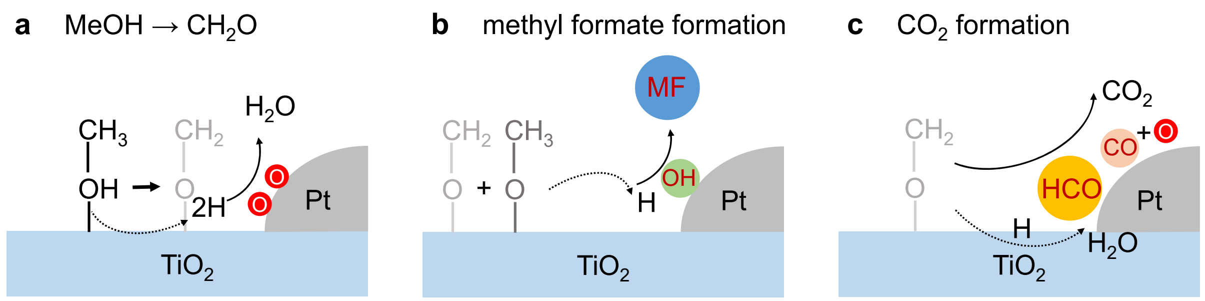

Supplementary Fig. 24 | Scheme of the reaction paths for methanol oxidation to **a** CH_2O intermediate, **b** methyl formate and **c** CO_2 formation.

25. Changed configuration of $*CH_2O$ on Pt nanorod on $TiO_2(110)$ and Pt(111)

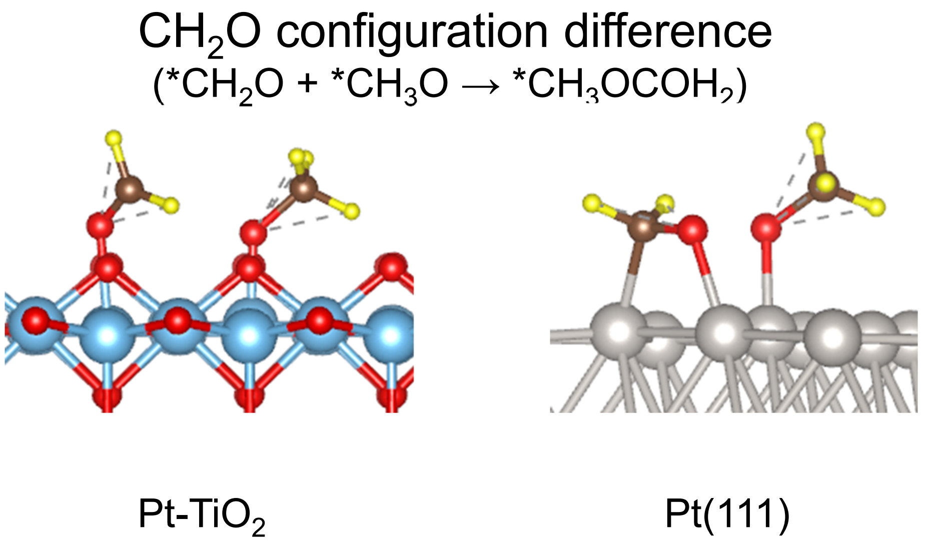

Supplementary Fig. 25 | CH_2O configuration difference. Optimized structures for $*CH_2O$ + $*CH_3O$ on Pt nanorod on $TiO_2(110)$ and Pt(111). The colors of each atom in the model are Pt (light grey), Ti (light blue), C(brown), O (red), H(yellow). The calculated configuration on Pt(111) was obtained from our previous work[2].

26. CO_2 adsorption energy on $TiO_2(110)$ in Pt nanorod/$TiO_2(110)$ model

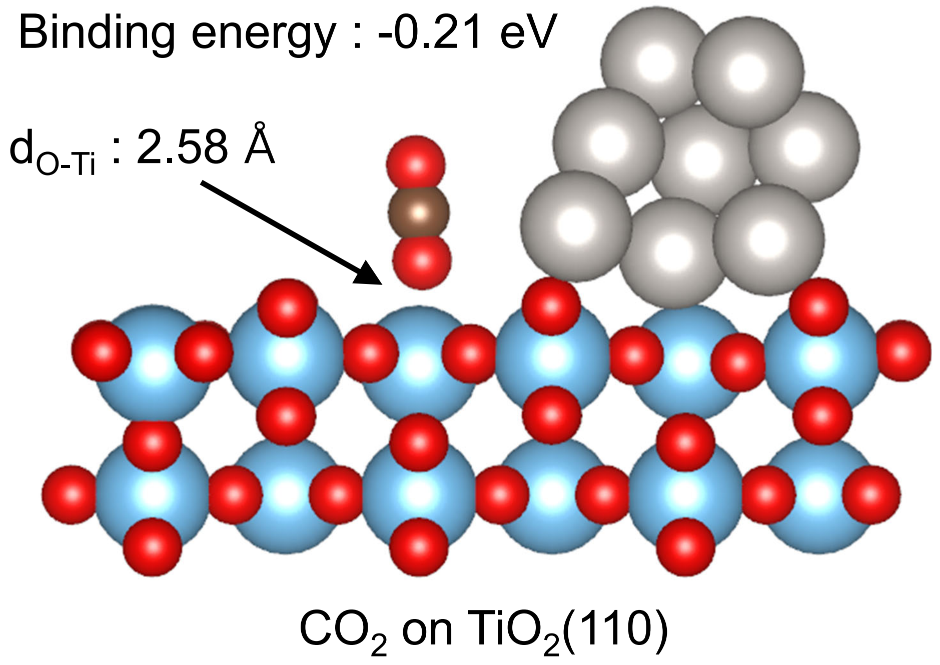

Supplementary Fig. 26 | Calculation of binding energy when CO_2 is adsorbed on $TiO_2(110)$ in Pt nanorod/$TiO_2(110)$ model by DFT.

Supplementary Tables

Pt film/TiO_2	1st cycle	2nd cycle	Average
Production of CO_2 (TOF)			
50 °C	0.036	0.047	0.042 ± 0.006
60 °C	0.137	0.119	0.128 ± 0.009
70 °C	0.365	0.341	0.353 ± 0.012
80 °C	0.997	0.773	0.885 ± 0.112
Production of methyl formate (TOF)			
50 °C	0.011	0.013	0.012 ± 0.001
60 °C	0.038	0.029	0.033 ± 0.004
70 °C	0.094	0.076	0.085 ± 0.009
80 °C	0.249	0.179	0.214 ± 0.035
Total TOF (molecules/site/s)			
50 °C	0.047	0.061	0.054 ± 0.007
60 °C	0.175	0.148	0.161 ± 0.014
70 °C	0.459	0.417	0.438 ± 0.021
80 °C	1.247	0.952	1.099 ± 0.148
Selectivity (%)			
50 °C	23.2	22.1	22.650 ± 0.550
60 °C	21.5	19.6	20.550 ± 0.950
70 °C	20.5	18.3	19.400 ± 1.100
80 °C	20.0	18.8	19.400 ± 0.600

Supplementary Table 1 | Measured catalytic activity and calculated selectivity on Pt film/TiO_2.

Pt nanowires/TiO_2	1st cycle	2nd cycle	Average
Production of CO_2 (TOF)			
50 °C	0.046	0.032	0.039 ± 0.007
60 °C	0.096	0.093	0.094 ± 0.002
70 °C	0.294	0.432	0.363 ± 0.069
80 °C	0.938	1.060	0.999 ± 0.061
Production of methyl formate (TOF)			
50 °C	0.030	0.020	0.025 ± 0.005
60 °C	0.042	0.046	0.044 ± 0.002
70 °C	0.113	0.182	0.148 ± 0.035
80 °C	0.363	0.397	0.380 ± 0.017
Total TOF (molecules/site/s)			
50 °C	0.076	0.052	0.064 ± 0.012
60 °C	0.138	0.139	0.138 ± 0.000
70 °C	0.407	0.614	0.511 ± 0.104
80 °C	1.301	1.457	1.379 ± 0.078
Selectivity (%)			
50 °C	39.2	38.0	38.600 ± 0.600
60 °C	30.3	33.2	31.750 ± 1.450
70 °C	27.7	29.7	28.700 ± 1.000
80 °C	27.9	27.3	27.600 ± 0.300

Supplementary Table 2 | Measured catalytic activity and calculated selectivity on Pt nanowires/TiO_2.

Type of nanodiode	Schottky barrier height (eV)
Pt film/TiO_2	0.87
Pt nanowires (width 15 nm)/TiO_2	1.02
Pt nanowires (width 20 nm)/TiO_2	0.85
Pt nanowires (width 50 nm)/TiO_2	0.89
TiO_2 nanowires (width 20 nm)/Pt	0.88

Supplementary Table 3 | Calculated Schottky barrier height for all Schottky nanodiodes by fitting *I-V* curve of each nanodevice to the thermionic emission theory.

	reaction pathway	reaction energy (eV)	barrier (eV)
	CH_3OH to form CH_3O		
1	$*CH_3OH \rightarrow *CH_3O+*H$	0.06	1.10
2	$*CH_3OH+*O \rightarrow *CH_3O+*OH$	-0.35	0.24
	CH_3O to form CH_2O		
3	$*CH_3O \rightarrow *CH_2O + *H$	0.40	0.59
4	$*CH_3O+*O \rightarrow *CH_2O + *OH$	−0.38	0.56
5	$*CH_3O+*OH \rightarrow *CH_2O + *H_2O$	−0.72	0.08
6	$*CH_3O+*CH_3O \rightarrow *CH_2O + *CH_3OH$	−0.25	0.73
	Formation of alkoxy hemiacetal		
7	$*CH_3O + *CH_2O \rightarrow *CH_3OCOH_2$	−0.07	0.00
	Alkoxy hemiacetal to form methylformate		
8	$*CH_3OCOH_2 \rightarrow CH_3OCOH_{(g)}+ *H$	0.08	0.84
9	$*CH_3OCOH_2 + *O \rightarrow CH_3OCOH_{(g)} + *OH$	−0.44	0.55
10	$*CH_3OCOH_2 + *OH \rightarrow CH_3OCOH_{(g)} + *H_2O$	−1.45	0.16
	CH_2O to form HCO		
11	$*CH_2O \rightarrow *HCO + *H$	0.02	0.63
12	$*CH_2O + *O \rightarrow *HCO + *OH$	−0.78	1.07
13	$*CH_2O+ *OH \rightarrow *HCO + *H_2O$	−1.48	0.56
	HCO to form CO		
14	$*HCO \rightarrow *CO + *H$	−0.05	0.48
15	$*HCO + *O \rightarrow *CO + *OH$	−1.25	0.56
16	$*HCO+ *OH \rightarrow *CO +*H_2O$	−1.73	0.59
	Formation of CO_2		
17	$*CO + *O \rightarrow CO_2$	−1.54	1.24

Supplementary Table 4 | Calculated reaction energies and barriers for possible reaction pathways for methanol oxidation on Pt nanorod on $TiO_2(110)$.

	reaction pathway	reaction energy (eV)	barrier (eV)
	CH_3OH to form CH_3O		
1	$*CH_3OH \rightarrow *CH_3O+*H$	0.20	0.65
2	$*CH_3OH+*O \rightarrow *CH_3O+*OH$	0.26	0.28
	CH_3O to form CH_2O		
3	$*CH_3O \rightarrow *CH_2O + *H$	−0.60	0.20
4	$*CH_3O+*O \rightarrow *CH_2O + *OH$	−0.38	0.81
5	$*CH_3O+*OH \rightarrow *CH_2O + *H_2O$	−1.25	0.42
6	$*CH_3O+*CH_3O \rightarrow *CH_2O + *CH_3OH$	−1.16	0.51
	Formation of alkoxy hemiacetal		
7	$*CH_3O + *CH_2O \rightarrow *CH_3OCOH_2$	−0.06	0.64
	Alkoxy hemiacetal to form methylformate		
8	$*CH_3OCOH_2 \rightarrow CH_3OCOH_{(g)}+ *H$	−1.75	1.89
9	$*CH_3OCOH_2 + *O \rightarrow CH_3OCOH_{(g)} + *OH$	−1.48	0.00
10	$*CH_3OCOH_2 + *OH \rightarrow CH_3OCOH_{(g)} + *H_2O$	−2.56	0.00
	CH_2O to form HCO		
11	$*CH_2O \rightarrow *HCO + *H$	−0.70	0.17
12	$*CH_2O + *O \rightarrow *HCO + *OH$	−0.99	0.25
13	$*CH_2O+ *OH \rightarrow *HCO + *H_2O$	−1.79	0.00
	HCO to form CO		
14	$*HCO \rightarrow *CO + *H$	−1.05	0.25
15	$*HCO + *O \rightarrow *CO + *OH$	−1.05	0.03
16	$*HCO+ *OH \rightarrow *CO +*H_2O$	−1.68	0.32
	Formation of CO_2		
17	$*CO + *O \rightarrow CO_2$	−0.61	0.86

Supplementary Table 5 | Calculated reaction energies and barriers for possible reaction pathways for methanol oxidation on Pt(111).

Supplementary References

1 Sze, S. M. & Ng, K. K. *Physics of semiconductor devices*. (John wiley & sons, 2006).

2 Lee, S. W. *et al.* Intrinsic Relation between Hot Electron Flux and Catalytic Selectivity during Methanol Oxidation. *ACS Catal.* **9**, 8424-8432 (2019).

3 Metiu, H., Chrétien, S., Hu, Z., Li, B. & Sun, X. Chemistry of Lewis acid–base pairs on oxide surfaces. *J. Phys. Chem. C* **116**, 10439-10450 (2012).

4 Bader, R. *Atoms in Molecules: A Quantum Theory* (Clarendon Press: Oxford England, 1994).

5 Hirunsit, P., Toyao, T., Siddiki, S. H., Shimizu, K. & Ehara, M. Origin of Nb_2O_5 Lewis acid catalysis for activation of carboxylic acids in the presence of a hard base. *Chemphyschem* **19**, 2848-2857 (2018).

6 Green, I. X., Tang, W., Neurock, M. & Yates, J. T. Spectroscopic observation of dual catalytic sites during oxidation of CO on a Au/TiO_2 catalyst. *Science* **333**, 736-739 (2011).

7 Green, I. X., Tang, W., Neurock, M. & Yates Jr, J. T. Low-temperature catalytic H_2 oxidation over Au nanoparticle/TiO_2 dual perimeter sites. *Angew. Chem. Int. Ed.* **50**, 10186-10189 (2011).
